# Health insurance coverage and timely antenatal care attendance in sub-Saharan Africa

**DOI:** 10.1186/s12913-022-07601-6

**Published:** 2022-02-11

**Authors:** Richard Gyan Aboagye, Joshua Okyere, Bright Opoku Ahinkorah, Abdul-Aziz Seidu, Betregiorgis Zegeye, Hubert Amu, Sanni Yaya

**Affiliations:** 1 Department of Family and Community Health, School of Public Health, University of Health and Allied Sciences, Ho, Ghana; 2 Department of Population and Health, University of Cape Coast, Cape Coast, Ghana; 3 School of Public Health, Faculty of Health, University of Technology Sydney, Sydney, Australia; 4 Centre for Gender and Advocacy, Takoradi Technical University, Takoradi, Ghana; 5 Department of Estate Management, Takoradi Technical University, Takoradi, Ghana; 6 College of Public Health, Medical and Veterinary Sciences, James Cook University, Townsville, Australia; 7 HaSET Maternal and Child Health Research Program, Shewarobit Field Office, Shewarobit, Ethiopia; 8 Department of Population and Behavioural Sciences, School of Public Health, University of Health and Allied Sciences, Hohoe, Ghana; 9 School of International Development and Global Studies, University of Ottawa, Ottawa, Canada; 10 The George Institute for Global Health, Imperial College London, London, United Kingdom

**Keywords:** National Health Insurance, Timely ANC, Pregnant women, Sub-Saharan Africa

## Abstract

**Introduction:**

Out-of-pocket payments for healthcare remain a significant health financing challenge in sub-Saharan Africa (SSA), preventing women from using maternal health services. There is a paucity of empirical literature on the influence of health insurance coverage on the timeliness of antenatal care (ANC) attendance in low- and middle-income countries. In this study, we examined the association between health insurance coverage and timely ANC attendance among pregnant women in SSA.

**Methods:**

Secondary data from Demographic and Health Surveys conducted between 2015 and 2020 in sixteen (16) sub-Saharan African countries with 113,918 women aged 15-49 years were included in the analysis. The outcome variable was the timing of antenatal care (ANC). A multilevel binary logistic regression analysis was carried out to determine the association between health insurance coverage and timely ANC.

**Results:**

The overall coverage of health insurance and timely antenatal attendance among pregnant women in SSA were 4.4% and 39.0% respectively. At the country level, the highest coverage of health insurance was found in Burundi (24.3%) and the lowest was in Benin (0.9%). For timely ANC attendance, the highest prevalence was in Liberia (72.4%) and the lowest was in Nigeria (24.2%). The results in the model showed that women who were covered by health insurance were more likely to have timely ANC attendance compared to those who were not covered by health insurance (aOR = 1.21, 95% CI = 1.11-1.31).

**Conclusion:**

Our findings show that that being covered under health insurance is associated with higher likelihood of seeking timely ANC attendance. To accelerate progress towards achievement of the Sustainable Development Goal targets by the year 2030, we recommend that governments and health insurance authorities across the sub-Saharan African countries actively implement health insurance policies as well as roll out health educational programmes that facilitate and ensure increased coverage of health insurance.

## Introduction

Achieving universal health coverage (UHC) is integral to the attainment of the Sustainable Development Goals (SDGs), particularly indicator 3.8.2 which seeks to identify people who spend greater than 10 or 25% of their household expenditure or income, respectively on health [1, 2]. UHC in this context denotes a situation where every individual and all communities have unparalleled access to quality health services without suffering any form of financial hardship [3]. This conceptualisation raises concerns about equity, quality, and financial risk protection [4]. Although the World Health Organisation (WHO) and the World Bank have been at the forefront of championing UHC due to the severe fiscal implications that are associated with UHC, its implementation has been largely driven by individual countries and states [5, 6]. Hence, different countries have adopted different mechanisms to arrive at UHC.

Prioritisation of health financing systems through health insurance schemes appears to be universal [4]. For example, countries such as China which has a population of over 1.3 billion people ensures health insurance coverage for its populace [7]. Several countries including the USA [8], Taiwan [9], and France [10] have all instituted national health insurance. In sub-Saharan Africa (SSA), countries like Ghana [3] and Nigeria [11] have health insurance schemes that aims to promote UHC and limit catastrophic health expenditure. Health insurance can either be a tax-financed government scheme, social health insurance, private compulsory health insurance and voluntary health insurance (i.e., private voluntary health insurance schemes and community-based health insurance) [12].

In most countries within SSA, health insurance offers greater opportunity for the population, especially, the vulnerable and those in poor economic status to have access to healthcare at any point in time [13]. One of such vulnerable populations that tends to benefit substantially from national health insurance coverage is pregnant women [13]. In some countries like Ghana, the government through an act of parliament (ACT 650) set up the national health insurance scheme in 2003, which exempts pregnant women from the payment of national health insurance premiums [14]. This is done as a means to encourage more pregnant women to enroll onto the national health insurance scheme [14]. Likewise, Nigeria in 2005 instituted their national health insurance scheme which offers 90% payment for the cost of drugs and healthcare while the subscriber paid only 10% of the cost of drugs [12].

Pregnant women are at risk of developing complications such as pregnancy-induced hypertension, anemia, severe persistent nausea, vomiting, and gestational diabetes [15]. Hence, antenatal care (ANC) becomes imperative to the health and wellbeing of the expectant mother and the unborn child [15]. For example, during ANC, pregnant women are provided with Intermittent preventive treatment in pregnancy [IPTp] for malaria control [16]. Moreover, through ANC attendance, the expectant mother and the unborn child benefit from nutrition and health checks, opportunity to detect high-risk pregnancy, counselling, and support for women and their families, as well as an increased propensity to have skilled birth delivery which may translate into a significant decline in the risk of maternal and neonatal mortality [17, 18].

Evidences from Ghana [19], Ethiopia [20], and Kenya [21] indicates that uptake of ANC is associated with maternal education, level of media exposure, region, place of residence, household wealth, place of delivery, marital status, age at first marriage, knowledge of ANC attendance, partner’s education and birth order. In relation to health insurance coverage, there is a plethora of evidence to suggest that health insurance coverage is significantly associated with ANC attendance among pregnant women [19, 22]. However, the question remains, to what extent does health insurance coverage influence not only the uptake of ANC attendance but more importantly, the timeliness of the attendance? This question remains less examined by the existing scholarship on health insurance coverage and ANC attendance. Timely ANC in this context refers to the ANC attendance for which the first contact occurred within the first 12 weeks (gestational period), with the subsequent ones occurring at 4-week intervals [23, 24]. Delaying ANC may result in serious deleterious consequences on the expectant mother and the unborn child, as well as exacerbate the risk of maternal morbidity and mortality [23, 24]. To address this concern of timeliness in ANC attendance, we examined the association between national health insurance coverage and timely ANC attendance among pregnant women in SSA.

## Methods

### Data source and study design

We analysed data from the most recent Demographic and Health Surveys (DHSs) of sixteen (16) countries in SSA. Countries were considered for inclusion into the study if they had dataset between 2015-2020 as well as have complete cases of variables of interest. In this study, the data were extracted from the women’s file (IR Recode) of the selected countries. The DHS is a nationally representative survey usually conducted periodically in over 85 low- and middle-income countries [25]. DHS employed a cross-sectional design in collecting data from the respondents. A two-stage sampling technique was employed to collect data from the respondents. Data on several health indicators such as maternal health care service utilisation, maternal and child health, nutrition, reproductive health, domestic violence, and men’s health was collected using a structured questionnaire [25]. The detailed survey methodology and sampling methods has been highlighted in a previous study [26]. A total of 113,918 women aged 15-49 years with complete observations were included in the analysis (Table 1). We relied on the Strengthening the Reporting of Observational Studies in Epidemiology (STROBE) statement in writing the manuscript [27]. The dataset is freely available for download at https://dhsprogram.com/data/available-datasets.cfm.

**Table 1:** Description of sample used in the study

Countries	Year of survey	Weighted N	Weighted %
1. Angola	2015-16	6,811	6.0
2. Benin	2017-18	7,932	7.0
3. Burundi	2016-17	8,831	7.7
4. Cameroon	2018	5,726	5.0
5. Ethiopia	2016	4,721	4.1
6. Gambia	2019-20	5,295	4.6
7. Guinea	2018	4,652	4.1
8. Liberia	2019-20	3,929	3.4
9. Mali	2018	5,237	4.6
10. Malawi	2015-16	13,168	11.6
11. Nigeria	2018	16,474	14.5
12. Sierra Leone	2019	7,147	6.3
13. Chad	2015	2,328	2.0
14. Uganda	2015	9,882	8.7
15. Zambia	2018	7,147	6.3
16. Zimbabwe	2015	4,638	4.1
**All countries**		**113,918**	**100.0**

### Study variables

#### Outcome variable

The outcome variable was the timing of antenatal care (ANC) attendance. The WHO defined the timing of ANC as the period in pregnancy during when the first ANC was sought [23]. To assess this variable, the women were asked﻿ “How many months pregnant were you when you first received ANC for this pregnancy? The response options to this question was recoded as ≤3 months or >3 months. The women with first ANC attendance in ≤3 months were categorised as having “timely ANC attendance” whilst those with ANC attendance in >3 months were said to have “late ANC attendance”. This categorisation has been used by studies that used the DHS dataset [20, 28, 29].

### Key explanatory variable

Health insurance coverage was the key explanatory variable. In assessing this variable, the women were asked the question “Are you covered by any health insurance?”. The response options were “No” and “Yes”. The use of health insurance coverage as a key explanatory variable was informed by studies that used the DHS dataset [14, 28].

### Covariates

A total of fifteen (15) variables were studied as covariates. The selection of the variables was informed by literature [20, 28–32] as well as their availability in the DHS datasets. The variables have been categorised into individual-level factors and contextual-level factors (*Household/community factor*).

#### Individual factors

The individual level factors were maternal age (15-19; 20-24; 25-29; 30-34; 35-39; 40-44; and 45-49), level of education (no education; primary; secondary; and higher), marital status (Never married; married; cohabiting; widowed; divorced; and separated), current working status (yes and no), religion (Christianity; Islamic; African Traditional; No religion; and Others), frequency of listening to radio (not at all; less than once a week, at least once a week; and almost every day), frequency of watching television (not at all; less than once a week, at least once a week; and almost every day), frequency of reading newspapers/magazines (not at all; less than once a week, at least once a week; and almost every day), getting medical help for self: permission to go (not a big problem and big problem), getting medical help for self: money to go (not a big problem and big problem), and getting medical help for self: distance to facility (not a big problem and big problem). Parity was recoded as “1”, “2”, “3”, and “4 or more”.

#### Household/community factors

Wealth index, place of residence, and studied countries were the contextual-level factors. From the DHS dataset, wealth index was coded as “poorest”, “poorer”, “middle”, “richer”, and “richest”. Sex of household head was coded as “male” and “female”. The age of household head was coded as “below 25”, “25-34”, “35-44”, and “45 and above”. Place of residence was coded as “urban” and “rural”. All 16 countries were included as a household/community level variables.

### Statistical analyses

**Fig. 1:**
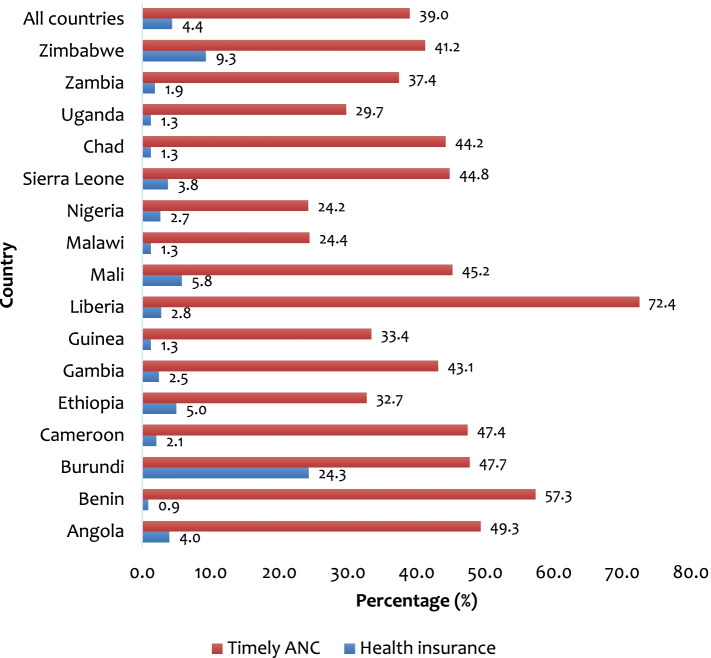
Coverage of health insurance and Timely ANC among pregnant women

We performed the data analysis using Stata software version 16.0 (Stata Corporation, College Station, TX, USA). First, percentages were used to present the results of the coverage of health insurance and timely ANC (Fig. 1). A Pearson chi-square test of independence was used to examine the relationship between health insurance, individual-level factors, contextual-level factors, and timely ANC (Table 2). All the variables that showed significance were placed in the regression model. We performed a multilevel binary logistic regression using four models to determine the association between health insurance and timely ANC controlling for the individual and household/community level factors (Table 3). We adopted multilevel regression analysis because of the complex structure of the DHS dataset [33]. Unlike the traditional regression, multilevel regression analysis best suites a hierarchical dataset structure similar to that of the DHS dataset, hence, its usage in the current study [34]. Also, the multilevel regression allowed us to incorporate variables measured at different levels of the hierarchy [34]. Model 0 showed the variance in timely ANC attributed to the clustering of the primary sampling units (PSUs) the health insurance and studied covariates. Model I was fitted to contain health insurance and individual-level factors. Model II contained the household/community-level factors. Model III was finally fitted to comprise of HEALTH INSURANCE, individual-level and household/community-level factors. We employed the Stata command “melogit” in fitting the four models. Akaike’s Information Criterion (AIC) tests for Model comparison was also used in the analysis. All the results were presented using adjusted odds ratios (aOR) at 95% Confidence Interval (CI). The women’s sample weight (v005/1,000,000) and the ‘svy’ command were used to correct for over and under-sampling, including the complex survey design to improve our findings’ generalisability. Hatt and Waters [35], argued that pooling data can reveal broader results that are “often obscured by the noise of individual data sets.” Therefore to calculate the pooled values an additional adjustment is needed to account for the variability in the number of individuals sampled in each country. This was achieved by using the weighting factor 1/(A*n_c_/n_t_), where A is the number of countries asked a particular question, n_c_ is the number of respondents for the country c, and n_t_ is the total number of respondents over all countries asked the question [36].

**Table 2:** Distribution of timely ANC among pregnant women in SSA across health insurance coverage and women’ socio-demographic characteristics

Variables	Weighted N	Weighted %	Timely ANC		
			Late (%)	Early (%)	***P***-value
**Covered by health insurance**					<0.001
No	108,870	95.6	61.6	38.4	
Yes	5,048	4.4	48.2	51.8	
**Maternal age**					<0.001
15-19	8,542	7.5	64.0	36.0	
20-24	26,060	22.9	60.7	39.3	
25-29	29,316	25.7	59.9	40.1	
30-34	23,137	20.3	60.8	39.2	
35-39	16,485	14.5	61.1	38.9	
40-44	7,762	6.8	62.4	37.6	
45-49	2,616	2.3	64.4	35.6	
**Maternal educational level**					<0.001
No education	38,458	33.8	62.5	37.5	
Primary	38,285	33.6	63.5	36.5	
Secondary	31,793	27.9	58.3	41.7	
Higher	5,382	4.7	49.6	50.4	
**Marital status**					<0.001
Never married	7,988	7.0	57.3	42.7	
Married	80,957	71.1	62.4	37.6	
Cohabiting	16,541	14.5	55.6	44.4	
Widowed	1,609	1.4	62.4	37.6	
Divorced	2,233	2.0	66.0	34.0	
Separated	4,590	4.0	59.9	40.1	
**Religion**					<0.001
Christianity	71,471.993	62.7	59.6	40.4	
Islamic	39,257.44	34.5	64.0	36.0	
African Traditional	1,062.8511	0.9	52.3	47.7	
No religion	1,646.23365	1.5	59.8	40.2	
Others	479	0.4	57.7	42.3	
**Maternal current working status**					0.006
No	37,090	32.6	61.8	38.2	
Yes	76,828	67.4	60.6	39.4	
**Parity**					<0.001
1	24,201.987	21.2	58.1	41.9	
2	21,617.456	19.0	58.9	41.1	
3	18,545.43	16.3	60.0	40.0	
4 or more	49,553.127	43.5	63.8	36.2	
**Getting medical help for self: Permission to go**					0.930
Not a big problem	96,033	84.3	61.0	39.0	
Big problem	17,885	15.7	61.1	38.9	
**Getting medical help for self: Distance to health facility**					<0.001
Not a big problem	70,682	62.0	59.7	40.3	
Big problem	43,236	38.0	63.2	36.8	
**Getting medical help for self: Getting money for treatment**					<0.001
Not a big problem	55,935	49.1	60.0	40.0	
Big problem	57,983	50.9	62.1	37.9	
**Frequency of reading newspaper/magazine**					<0.001
Not at all	98,259	86.2	61.8	38.2	
Less than once a week	9,362	8.2	58.5	41.5	
At least once a week	5,887	5.2	53.4	46.6	
Almost every day	410	0.4	39.7	60.3	
**Frequency of listening to radio**					<0.001
Not at all	51,492	45.2	62.8	37.2	
Less than once a week	23,169	20.4	60.4	39.6	
At least once a week	36,257	31.8	60.0	40.0	
Almost every day	3,000	2.6	49.7	50.3	
**Frequency of watching television**					<0.001
Not at all	70,118.479	61.6	63.7	36.3	
Less than once a week	14,500.483	12.7	60.3	39.7	
At least once a week	24,303.322	21.3	56.1	43.9	
Almost every day	4,995.7151	4.4	50.4	49.6	
**Wealth index**					<0.001
Poorest	21,819	19.2	64.1	35.9	
Poorer	23,179	20.3	63.9	36.1	
Middle	23,137	20.3	63.5	36.5	
Richer	23,432	20.6	60.8	39.2	
Richest	22,351	19.6	52.7	47.3	
**Sex of household head**					<0.001
Male	89,888	78.9	61.6	38.4	
Female	24,030	21.1	59.1	40.9	
**Age of household head**					0.025
Below 25	7,360	6.5	62.3	37.7	
25-34	35,692	31.3	60.3	39.7	
35-44	35,321	31.0	61.5	38.5	
45 and above	35,545	31.2	61.0	39.0	
**Residence**					<0.001
Urban	41,091	36.1	57.0	43.0	
Rural	72,827	63.9	63.3	36.7	

*p-values obtained from Pearson chi-square test

**Table 3:** Fixed and random effect analysis of association between health insurance and timely ANC among pregnant women in sub-Saharan Africa

Variable	Model 0	Model I aOR [95% CI]	Model II aOR [95% CI]	Model III aOR [95% CI]
**Fixed effect results**				
**Covered by health insurance**				
No		1 [1.00,1.00]		1 [1.00,1.00]
Yes		1.51^***^ [1.40,1.64]		1.21^***^ [1.11,1.31]
**Maternal age**				
15-19		1 [1.00,1.00]		1 [1.00,1.00]
20-24		1.23^***^ [1.15,1.31]		1.22^***^ [1.14,1.30]
25-29		1.42^***^ [1.32,1.54]		1.34^***^ [1.24,1.46]
30-34		1.50^***^ [1.38,1.63]		1.42^***^ [1.30,1.54]
35-39		1.56^***^ [1.43,1.70]		1.46^***^ [1.33,1.60]
40-44		1.51^***^ [1.37,1.67]		1.43^***^ [1.29,1.58]
45-49		1.44^***^ [1.28,1.64]		1.39^***^ [1.22,1.59]
**Maternal educational level**				
No education		1 [1.00,1.00]		1 [1.00,1.00]
Primary		0.90^***^ [0.86,0.94]		1.17^***^ [1.13,1.23]
Secondary		0.97 [0.92,1.02]		1.18^***^ [1.12,1.24]
Higher		1.14^**^ [1.04,1.25]		1.56^***^ [1.41,1.72]
**Marital status**				
Never married		1 [1.00,1.00]		1 [1.00,1.00]
Married		0.96 [0.89,1.03]		1.34^***^ [1.25,1.45]
Cohabiting		1.16^***^ [1.07,1.25]		1.25^***^ [1.15,1.36]
Widowed		0.99 [0.86,1.14]		1.35^***^ [1.17,1.56]
Divorced		0.82^**^ [0.72,0.93]		1.32^***^ [1.16,1.51]
Separated		1.02 [0.92,1.14]		1.35^***^ [1.22,1.49]
**Religion**				
Others		1 [1.00,1.00]		1 [1.00,1.00]
Christianity		0.90 [0.72,1.12]		1.01 [0.81,1.25]
Islamic		0.77^*^ [0.61,0.96]		0.84 [0.67,1.06]
African Traditional		1.30 [0.98,1.72]		0.82 [0.62,1.09]
No religion		0.85 [0.65,1.11]		0.77^*^ [0.59,1.00]
**Maternal current working status**				
No		1 [1.00,1.00]		1 [1.00,1.00]
Yes		1.05^*^ [1.01,1.09]		1.00 [0.96,1.04]
**Parity**				
1		1 [1.00,1.00]		1 [1.00,1.00]
2		0.88^***^ [0.83,0.92]		0.86^***^ [0.82,0.91]
3		0.79^***^ [0.75,0.84]		0.79^***^ [0.74,0.84]
4 or more		0.66^***^ [0.62,0.70]		0.68^***^ [0.64,0.73]
**Getting medical help for self: Getting money for treatment**				
Not a big problem		1 [1.00,1.00]		1 [1.00,1.00]
Big problem		1.00 [0.96,1.04]		0.96 [0.93,1.00]
**Getting medical help for self: Distance to health facility**				
Not a big problem		1 [1.00,1.00]		1 [1.00,1.00]
Big problem		0.92^***^ [0.89,0.96]		0.98 [0.94,1.02]
**Frequency of reading newspaper/magazine**				
Not at all		1 [1.00,1.00]		1 [1.00,1.00]
Less than once a week		0.97 [0.92,1.03]		1.07^*^ [1.01,1.13]
At least once a week		1.07 [0.98,1.17]		1.14^**^ [1.05,1.24]
Almost every day		1.42^*^ [1.06,1.91]		1.31 [1.00,1.74]
**Frequency of listening to radio**				
Not at all		1 [1.00,1.00]		1 [1.00,1.00]
Less than once a week		1.04 [0.99,1.09]		1.02 [0.97,1.07]
At least once a week		1.01 [0.97,1.05]		1.02 [0.98,1.07]
Almost every day		1.25^**^ [1.09,1.43]		1.21^**^ [1.06,1.38]
**Frequency of watching television**				
Not at all		1 [1.00,1.00]		1 [1.00,1.00]
Less than once a week		1.13^***^ [1.07,1.19]		1.06^*^ [1.00,1.12]
At least once a week		1.23^***^ [1.17,1.30]		1.11^***^ [1.05,1.18]
Almost every day		1.28^***^ [1.14,1.44]		1.07 [0.95,1.21]
**Wealth index**				
Poorest			1 [1.00,1.00]	1 [1.00,1.00]
Poorer			1.02 [0.97,1.07]	0.98 [0.93,1.03]
Middle			1.05^*^ [1.00,1.11]	0.97 [0.92,1.02]
Richer			1.22^***^ [1.15,1.29]	1.05 [0.99,1.12]
Richest			1.85^***^ [1.73,1.98]	1.36^***^ [1.26,1.47]
**Sex of household head**				
Male			1 [1.00,1.00]	1 [1.00,1.00]
Female			1.03 [0.99,1.07]	1.03 [0.99,1.08]
**Age of household head (years)**				
Below 25			1 [1.00,1.00]	1 [1.00,1.00]
25-34			1.01 [0.95,1.09]	1.02 [0.95,1.10]
35-44			0.95 [0.89,1.01]	1.00 [0.93,1.07]
45 and above			0.92^*^ [0.86,0.99]	0.99 [0.91,1.06]
**Residence**				
Urban			1 [1.00,1.00]	1 [1.00,1.00]
Rural			1.14^***^ [1.08,1.20]	1.18^***^ [1.12,1.25]
**Countries**				
Angola			1 [1.00,1.00]	1 [1.00,1.00]
Benin			1.38^***^ [1.24,1.54]	1.58^***^ [1.41,1.78]
Burundi			0.90 [0.80,1.02]	0.91 [0.80,1.03]
Cameroon			0.94 [0.83,1.07]	0.96 [0.84,1.09]
Ethiopia			0.47^***^ [0.41,0.55]	0.50^***^ [0.43,0.58]
Gambia			0.86^*^ [0.74,0.99]	0.94 [0.80,1.10]
Guinea			0.51^***^ [0.44,0.60]	0.63^***^ [0.53,0.74]
Liberia			2.86^***^ [2.50,3.28]	3.15^**^ [2.74,3.62]
Mali			0.82^**^ [0.73,0.94]	0.99 [0.86,1.14]
Malawi			0.32^***^ [0.28,0.36]	0.31^***^ [0.28,0.35]
Nigeria			0.31^***^ [0.28,0.35]	0.31^***^ [0.28,0.35]
Sierra Leone			0.86^*^ [0.76,0.97]	1.02 [0.89,1.17]
Chad			0.79^**^ [0.68,0.91]	0.95 [0.82,1.11]
Uganda			0.42^***^ [0.38,0.47]	0.42^***^ [0.37,0.47]
Zambia			0.62^***^ [0.55,0.70]	0.56^***^ [0.50,0.64]
Zimbabwe			0.70^***^ [0.62,0.79]	0.61^***^ [0.53,0.69]
**Random effects**				
PSU variance (95% CI)	0.196 [0.165, 0.232]	0.188 [0.159, 0.224]	0.089 [0.072, 0.110]	0.080 [0.066, 0.098]
ICC	0.056	0.054	0 .026	0.024
Wald chi-square	Reference	782.93 (<0.001)	2788.42 (<0.001)	3568.39 (<0.001)
**Model fitness**				
Log-likelihood	-75547.51	-74627.774	-71913.807	-71399.091
AIC	151099	149327.5	143879.6	142918.2
N	113918	113918	113918	113918
Number of clusters	1,397	1,397	1,397	1,397

Exponentiated coefficients, 95% confidence intervals in brackets, *aOR* adjusted odds ratios, *CI* Confidence Interval, * *p* < 0.05, ** *p* < 0.01, *** *p* < 0.001, 1 Reference, category *PSU* Primary Sampling Unit, *ICC* Intra-Class Correlation, *AIC* Akaike’s Information Criterion

### Ethical considerations

Ethical approval was not sought for this study since the data is freely available in the public domain. From the DHS reports, ethical clearance was sought and all ethical guidelines governing the use of human subjects in research were strictly adhered to. The detailed ethical guidelines are available at http://goo.gl/ny8T6X.

### Availability of data and materials

Data for this study is available at DHS dataset, http://dhsprogram.com/data/available-datasets.cfm

## Results

### Health insurance coverage and timely antenatal care attendance among pregnant women in SSA

The overall coverage of health insurance and timely ANC attendance among pregnant women in SSA were 4.4% and 39.0% respectively. At the country level, the highest coverage of health insurance was found in Burundi (24.3%) with the lowest coverage in Benin (0.9%). For timely ANC attendance, the highest prevalence was in Liberia (72.4%) and the lowest in Nigeria (24.2%) (Fig. 1).

### Bivariable analysis of health insurance coverage and timely antenatal care attendance among pregnant women in SSA

Table 2 presents the results on the distribution of timely ANC among pregnant women in SSA across health insurance coverage and women’ socio-demographic characteristics. With health insurance coverage, the results showed significant differences across timing of ANC at p<0.005. Specifically, timely ANC was higher among pregnant women who were covered by health insurance (51.8%) compared to those who were not covered by health insurance (38.4%). All the socio-demographic characteristics of pregnant women also showed significant differences in the timing of ANC, except getting medical help for self: permission to go.

### Multilevel regression analysis of health insurance coverage and timely antenatal care attendance among pregnant women in SSA

#### Measure of association (Fixed effect results)

Table 3 shows results of the multilevel logistic regression analysis of the association between health insurance coverage and timely antenatal care attendance among pregnant women in SSA while controlling for significant covariates. The last model (Model III), which contained health insurance and all the covariates was considered the best-fit model (AIC=142918.2). The results in the model showed that women who were covered by health insurance were more likely to have timely ANC attendance compared to those who were not covered by health insurance (aOR = 1.21, 95% CI = 1.11-1.31).

With the covariates, timely ANC was higher among pregnant women of all age categories compared to women aged 15-19. Compared to pregnant women with no formal education, those with at least primary education were more likely to have timely ANC attendance, with the highest odds among those with higher education (aOR=1.56, 95% CI= 1.41,1.72). Women of all statuses of marriage were more likely to have timely ANC attendance compared to never married pregnant women. Pregnant women who were exposed to newspaper/magazine, radio, and television were more likely to have timely ANC attendance compared to those who were not exposed to any of these media sources. A higher likelihood of timely ANC attendance was found among pregnant women of the richest wealth index compared to those of the poorest wealth index (aOR = 1.36, 95% CI = 1.26-1.47). Pregnant women who lived in rural areas were more likely to go for ANC attendance on time compared to those who lived in urban areas (aOR = 1.18, 95% CI = 1.12-1.25). The odds of timely ANC decreased with parity with women who had four or more children having the lowest odds of timely ANC attendance (aOR = 0.68, 95% CI = 0.64-0.73).

#### Measure of association (Random effect results)

The results from Table 3 showed a minute variation in timely ANC across the PSUs in the empty model (Model O) [σ2 =0.196; 95% (CI =0.165–0.232)]. In the Intra-Class Correlation (ICC), an approximately 6% (0.056) of the total variance was attributed to the between clusters differences of the communities were the pregnant women resided. The ICC decreased from about 5% (0.054) in Model I to 2% (0.024) in Model III. Hence, the study observed a decrease in variation across all the models attributable to the residential communities of the pregnant women. Also, the AIC values showed a significant reduction from 151099 in Model O to 142918.2 in Model III. As a result, Model III was adopted as the best fitted model for the study.

## Discussion

Overall, coverage of health insurance and timely ANC attendance was low, at 4.4 and 39.0 percent respectively. This low prevalence observed in our study poses a serious public health concern and a likely staller for attaining the SDG target 3.8.2. While the general prevalence of timely ANC attendance and coverage of health insurance was low, our study observed that there was some level of heterogeneity with respect to inter-country variations. For example, Liberia had the highest prevalence of timely ANC attendance (72.4%) whereas Burundi recorded the highest coverage of health insurance (24.3%). Contextual evidence from Liberia reveals that maternal health care has been improving substantially since the end of the Liberian civil war in 1999, and is evident in the timing of the first ANC attendance [37]. Thus, explaining the high level of timely ANC attendance as observed in the present study. In the case of Burundi, the government with external support from implementation partners provided a complete exemption for the cost of healthcare services for pregnant women in 2006 [38]. This could explain the relatively high coverage of health insurance in Burundi as compared to a country like Ghana that implemented free maternal health care later in 2008.

The results from this study also revealed that health insurance coverage was significantly associated with timely ANC attendance among pregnant women as those covered by the health insurance having a higher likelihood of reporting timely ANC attendance. This finding corroborates earlier studies [37] that have found health insurance coverage to have a significant association with the odds of reporting timely ANC attendance. Previous studies [19, 22] have found that health insurance coverage is quintessential in eliminating out-of-pocket payments and significantly removing the financial barriers that often limit women’s capacity to attend ANC on time. Thus, serving as a motivating factor that encourages pregnant women to seek timey ANC attendance.

Education emerged as a significant covariate in our analyses. Pregnant women with no formal education were less likely to have timely ANC attendance as compared to those who had formal education. Our study further shows that among pregnant women with formal education, those who had secondary or higher education tend to have a higher likelihood of reporting timely ANC attendance as compared to their counterparts who have only primary education. The result is substantiated by previous studies from Ghana [19], Ethiopia [20], and Kenya [21] which found a higher likelihood in the uptake and timeliness of ANC attendance. A plausible explanation to this finding could be that women who have secondary or higher education are more likely to be exposed to the mass media which serves as a conduit for educating women, particularly about the relevance of attending ANC on time as well as the dangers of delaying ANC attendance. Another justifiable reason for this finding could be that pregnant women with higher levels of education tend to be empowered and are therefore able to take critical decisions concerning their reproductive health in an autonomous manner. Hence, the likelihood of delaying ANC attendance is significantly reduced while increasing the odds of timely ANC attendance. This also explains why women who were exposed to the mass media (i.e., newspaper/magazine, radio, and television) were more likely to have timely ANC attendance.

Compared to unmarried pregnant women, married pregnant women were more likely to have timely ANC attendance. An analogous finding was reported by Sakeah et al. [19]. This result is best explained from the socio-cultural perspective. In most courtiers within the sub-Saharan African region, pregnancy out of wedlock is taboo and is further exacerbated by the religious indoctrinations that portray pregnancy out of wedlock as sinful [39, 40]. As such, unmarried women who get pregnant find it difficult to attend ANC, and those that gather the courage to attend ANC often delay [39, 40]. This delay comes as a defensive mechanism to avoid stigma and ridicule from society as well as from healthcare providers [39, 40]. Another plausible explanation could be that unmarried women may sometimes find it difficult to access the financial resource needed to seek timely ANC attendance , particularly in areas where national health coverage is extremely low and there is no complete exemption for the cost of healthcare to pregnant women [19].

After controlling for other variables, pregnant women in rural areas were more likely to have timely ANC attendance. This finding is contrary to that of previous studies that higher risk of delayed initiation of ANC among rural dwelling women as compared to urban women, mainly due to the presence of substantial barriers in rural communities, such as issues of long-distance to health care facilities and unavailability of health facilities [19, 20]. Plausibly, this could be attributed to the busy and strenuous work demands of urban-dwelling women which makes it difficult for them to initiate ANC visits early as compared to women residing in rural settlements.

We also found a higher likelihood of timely ANC attendance among pregnant women of the richest wealth index compared to those of the poorest wealth index. The poorest women tend to suffer several challenges with respect to being able to afford health care as well as being capable of making decisions concerning their reproductive health and health promotive behaviors [41]. Hence, making them less likely to seek timely ANC attendance. Our study also revealed that the odds of timely ANC attendance decrease with higher parity. Multiparous women the lowest odds of timely ANC attendance. This finding confirms a related study conducted in Ethiopia [42] that found multiparous women to have the lowest likelihood of achieving timely ANC attendance. Given that multiparous women have greater experience with childbirth, they are likely to feel more confident about handling their pregnancy [42]. As such, they are likely to have lower perception about the relevance of ANC compared with primiparous women who are often inexperienced and will, therefore, feel inadequate to handle their pregnancy [42]. Hence, uniparous women have greater tendency to have timely ANC attendance.

### Strengths and limitations

Nationally representative data were employed to examine the association between health insurance coverage and timely ANC attendance among pregnant women. This provided a wider coverage for our study that makes the findings statistically generalisable to the 16 sub-Saharan African countries included in the study. Moreover, we used robust analytical tools that make our study replicable and valid. Nevertheless, some inherent limitations must be taken into consideration during the interpretation of our findings. Due to the cross-sectional nature of the dataset used, causal inference cannot be established. Also, the data was self-reported, hence, there is the possibility of recall bias and social desirability.

## Conclusion

Based on the findings from our study, we conclude that being covered by health insurance is associated with a higher likelihood of seeking timely ANC attendance. For this reason, we recommend that governments and national health insurance authorities across the sub-Saharan African countries to actively implement health insurance policies as well as roll out health educational programs that will facilitate and ensure high coverage of health insurance. Moreover, our study shows that higher level of education, wealth index, and urban residency are facilitating factors for timely ANC attendance. Therefore, it is imperative for the various governments across the sub-region to invest intensively in female education as well as improving the socio-economic status of women. This will ensure greater success in Africa’s quest to eliminate delayed ANC attendance, and rather promote timely attendance. Given that exposure to the mass media is an important factor in influencing timely ANC, attendance we recommend that the TV, radio, and newspapers be used as a conduit of delivering critical, accurate information and education about the importance of seeking timely ANC attendance and the potential dangers of delaying ANC.

## Data Availability

Data for this study is available at: http://dhsprogram.com/data/available-datasets.cfm

## Abbreviations

ANC: Antenatal Care
aOR: Adjusted Odds Ratio
DHSs: Demographic and Health Surveys
NHI: National Health Insurance
SDGs: Sustainable Development Goals
SSA: Sub-Saharan Africa
STROBE: Strengthening the Reporting of Observational Studies in Epidemiology
WHO: World Health Organization
